# Distinguishing Different Emotions Evoked by Music via Electroencephalographic Signals

**DOI:** 10.1155/2019/3191903

**Published:** 2019-03-06

**Authors:** Yimin Hou, Shuaiqi Chen

**Affiliations:** ^1^School of Automation Engineering, Northeast Electric Power University, Jilin, China; ^2^Luneng New Energy (Group) Co., Beijing, China

## Abstract

Music can evoke a variety of emotions, which may be manifested by distinct signals on the electroencephalogram (EEG). Many previous studies have examined the associations between specific aspects of music, including the subjective emotions aroused, and EEG signal features. However, no study has comprehensively examined music-related EEG features and selected those with the strongest potential for discriminating emotions. So, this paper conducted a series of experiments to identify the most influential EEG features induced by music evoking different emotions (calm, joy, sad, and angry). We extracted 27-dimensional features from each of 12 electrode positions then used correlation-based feature selection method to identify the feature set most strongly related to the original features but with lowest redundancy. Several classifiers, including Support Vector Machine (SVM), C4.5, LDA, and BPNN, were then used to test the recognition accuracy of the original and selected feature sets. Finally, results are analyzed in detail and the relationships between selected feature set and human emotions are shown clearly. Through the classification results of 10 random examinations, it could be concluded that the selected feature sets of Pz are more effective than other features when using as the key feature set to classify human emotion statues.

## 1. Introduction

Recognition of emotion state is an important aim for the development of advanced brain-computer interfaces (BCIs). For this application, emotion recognition using the electroencephalogram (EEG) has garnered widespread interest due to the convenience, high resolution, and reliability of EEG recordings. Music can evoke powerful emotions, and these emotions are associated with distinct EEG signal patterns. Identification of the EEG signals associated with these emotions may help elucidate the neurological basis for music appreciation, contribute to the development of music programmes for mood therapy [1], and provide biomarkers and novel methods for studying neuropsychiatric disorders such as depression and Alzheimer's disease [2].

Numerous studies have identified EEG signals associated with distinct features of music, including familiarity, level of processing, phrase rhythm, and subjective emotional response. Thammasan et al. extracted power density spectra and fractal dimensions from the Database for Emotion Analysis using Physiological Signals (DEAPs) and found that using low familiarity music improved the accuracy of recognition regardless of whether the classifier was support vector machine (SVM), multilayer perception, or C4.5 [3]. Kumagai et al. investigated the relationship between cortical response and familiarity of music. They found that the two peaks of the cross-correlation values were significantly larger when listening to unfamiliar or scrambled music compared with familiar music. Collectively, these findings suggest that the cortical response to unfamiliar music is stronger than that to familiar music and so is more appreciate for classification applications by BCIs [4]. Santosa et al. design series of experiments, in which, different level noise, such as no noise (NN), midlevel noise (MN), and high-level noise (HN), was added into the music. The 14 subjects were tested in four different auditory environments: music segments only, noise segments only, music + noise segments, and the entire music interfered by noise segments. Then, their responses data were analyzed to determine the effects of background noise on the hemispheric lateralization in music processing [5]. Hong and Santosa investigated whether activations in the auditory cortex caused by different sounds can be distinguished using functional near-infrared spectroscopy (fNIRS) [6]. Bigliass et al. used musical stimuli as interference to examine how the brain controls action when processing two tasks at the same time [7]. Lopata et al. reported stronger activity of the frontal lobe alpha band, which is correlated with the mental imagery of music after it has been played and studied, in subjects with music improvisation training compared with subjects without training. Thus, level of processing (e.g., creative processing) influences the EEG response. Moreover, these results suggest that musical creativity can be learned and improved and that these changes are measurable by EEG [8]. Phneah and Nisar compared the EEG responses induced by favourite subject-selected music to “relaxing” music selected based on alpha wave binaural beats and found that the relaxing music had stronger and longer-lasting physiological and psychological soothing effects [9]. Thus, EEG signals can help in the selection of mood-modifying music. Bridwell et al. compared the EEG responses evoked by structured guitar notes and random notes and found a waveform at 200 ms that was more strongly activated by the structured sequence. Further, the result of this study is that 4 Hz note patterns appears somewhat distinct from the sensitivity of statistical regularities of “auditory oddball” [10]. Martínez-Rodrigo et al. found distinct EEG responses in theta and alpha bands to phrase rhythm variations of two classical sonatas, one in bipartite form and the other in rondo form [11]. Zhou et al. compared the processing of musical meaning conveyed by direction of pitch change in congenital amusia. Twelve Mandarin-speaking amusia and 12 controls performed a recognition (implicit) and a semantic congruency judgement (explicit) task while their EEG waveforms were recorded. The authors concluded that amusia are able to process iconic musical meaning through multiple acoustic cues in natural musical excerpts but not through the direction of pitch change [12]. Many researchers studied the relationships between human emotions and their EEG signal. Lu et al. selected nine musical passages as stimuli and divided them into three groups using variance test and *t*-test according to the two-dimensional model of emotion. To analyse EEG signals, they extracted the power density spectra of different bands and used principle component analysis (PCA) dimensionality reduction for feature selection. They found that emotion recognition accuracy by SVM was higher using the average power information of beta and gamma bands compared with other bands. Thus, beta and gamma bands appear to contain signal information useful for emotional discrimination [13]. Di et al. presented an analysis procedure in order to study the affect of human emotion using EEG characteristics induced by sound stimuli with different frequencies [14]. Kurbalija et al. proposed a method of emotion recognition from EEG signals using distance-based time-series classification. The results showed that the EEG signal could be used successfully to construct models for recognition of emotions, individuals, and other related tasks [15]. Kobayashi and Nakagawa presented the emotion fractal analysis method (EFAM) in their paper. They assessed emotions based on EEG data to propose a BCI system which can recognize emotions including delight, anger, sorrow, and pleasure and used that information to manipulate an electric wheelchair [16]. Zhao et al. sampled the EEG signal of the volunteers when they watched affective films. After extracting the EEG features, SVM was employed as the classifier to recognize human emotions [17]. Ivanovic et al. collected EEG data from ten males between 25 and 40 years old and presented an automatic real-time classification algorithm using EEG data cited by self-induced emotions [18]. Yoon and Chung proposed a Bayes' theorem-based classifier which used supervised learning algorithm to recognize human emotion according to the volunteers' EEG data. In the recognition, Fast Fourier Transform (FFT) analysis was used for feature extraction [19].

The complexity of EEG signals has hampered the selection of optimal feature sets for discrimination of emotions evoked by music. To compensate for the lack of single feature recognition, many previous studies have attempted to extract one or several linear or nonlinear dynamic characteristics of EEG signals for distinguish different music stimuli by machine learning. For example, to study and prevent mild depression, Li et al. extracted 816 features (17 features of 3 frequency bands from 16 electrodes) and improved the recognition rate using CFS dimension reduction and machine learning. They also suggested that signals from electrode positions FP1, FP2, F3, O2, and T3 are most strongly related to mild depression [20]. Xu and Zuo proposed an algorithm based on mutual information and PCA to compensate for the lack of nonlinear relationships between the features of PCA alone using the database of the 2005 international BCI competition. After joint power spectrum estimation, continuous wavelet analysis, wavelet package analysis, and Hjorth parameter calculations, they used the newly proposed algorithm for selection and compared results to the PCA algorithm alone. They found that dimension reduction by the proposed algorithm improved classification accuracy using SCM as the classifier [21].

Though many achievements have been gained in this field, there are still some problems for emotion recognition. Firstly, the main results in emotional recognition focus on visual emotion recognition. The researches in recognition of emotion evoked by music are less and the recognition rates are lower. But in human daily life, music-induced emotions are effective and persistent, allowing a clearer observation of brain activity in this emotional state [22]. Secondly, the spatial resolution of EEG signal is low. So, the effect of the study of brain cognitive rules based on features is poor; most of them can only be precise to a certain brain area, but it is impossible to put forward more precise brain cognitive mechanism. The effectiveness of the study of brain cognitive rules based on features is unsatisfactory. Most of them can only be located to a certain brain area, but it is difficult to construct the relationships between EEG electrode and emotions' classification [23]. Lastly, due to the complexity of the EEG, most previous studies on the relationships between EEG signal features and music-evoked emotions have focused on the analysis of a few specific characteristics. However, few studies have conducted a comprehensive unbiased analysis of whole-brain EEG signals associated with music-evoked emotions and then selected those with highest discriminative power for various classifiers [24].

In the presented study, we want to obtain the most influential EEG signal feature set of the human emotion classification. To achieve this goal, 18-dimension linear features and 9-dimension nonlinear features were extracted for every electrode, and the correlation-based feature selection (CFS) method was employed to select the influential feature set. To verify the influence of the selected feature set, the classification methods including BPNN, SVM, C4.5, and LDA were used in the procedure of human emotion classification. The experiment results showed that the selected feature set of Pz electrode and the classification method C4.5 were more effective in human emotion recognition.

## 2. Methods

**Figure 1:**
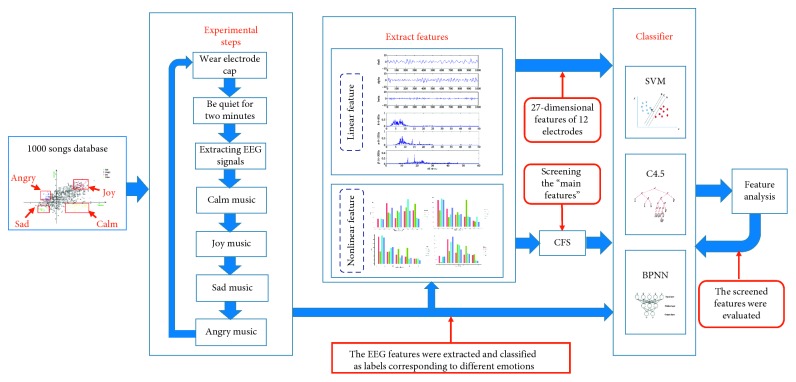
Study flow chart.

As shown in Figure 1, there were five stages in this study: collection of volunteers' subject data, EEG recording during listening music, extraction of EEG features, emotion classification, and detailed analysis for validation, dimension reduction, and accuracy improvement.

### 2.1. Source of Music Stimuli

**Figure 2:**
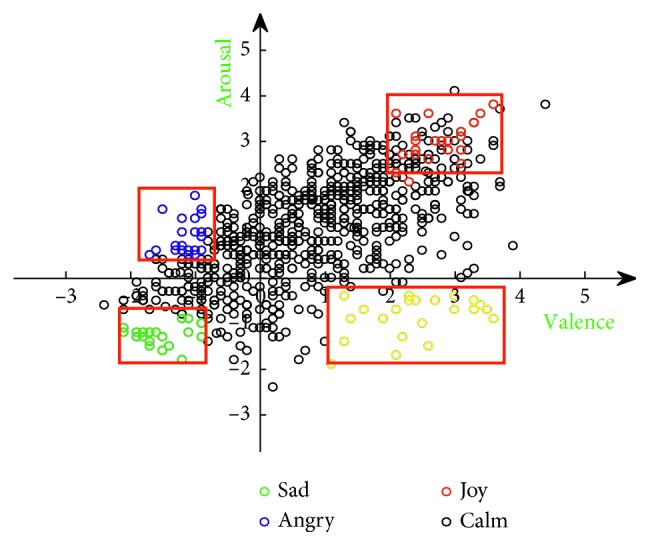
Emotional classification of the sample music.

The music stimuli used in this study are from the database of 1000 songs, which was selected by Mohammad Soleymani and colleagues of the University of Geneva for emotion analysis from the Free Music Archive (http://freemusicarchive.org/). For each song, the sampling rate in the database is 44,100 Hz and the length is 45 s. Each song is also marked with the arousal dimension and the valence dimension for classification by the two-dimensional model [25, 26]. For classification, we separated the data into four groups according to the highest average scores and variance and there were 22 samples in “joy” and “calm” group, while 20 samples in “sad” and “angry” group. To balance the data number, 20 music samples were chosen for every emotion. The average scores, variance of arousal, and valence values were provided by the above database. As shown in Figure 2, there was no significant difference between the scores in different groups by checking the correlation between the groups using analysis of variance (ANOVA). As shown in Table 1, scores in arousal dimension of sad and calm are same; scores in valence dimension of angry and calm are same. So, we just compared 12 pairs in the table which showed the homogeneity test of variance. Then, according to the reviewer's suggestion, we used the analysis of variance in Table 2. It is showed that the *P* value is all less than 0.05, indicating that there are significant differences in the four emotional statues in valence and arousal dimension. Four kinds of emotions can be regarded as different kinds of data and used as different stimulus to cause EEG signals of audiences in experiments [27]. In the experiment, the data with highest scores for both arousal and valence dimension were defined as “happy” or “joy,” that with lowest scores for the two dimensions were defined as “sad,” and that with highest arousal and lowest valence as “angry” and defined the data as “calm” in the opposite case.

### 2.2. EEG Recording

The participants were eight graduate students who have not music background (23.11 ± 3.14 years old, six males and two females). Before the experiment, the subjects provided personal information and informed consent. They were requested to avoid stimuli and refrain from activities that may induce strong emotions before one day of the experiment. At the beginning of the experiment, EEG electrodes were applied, and subjects were requested to remain calm with eyes closed for 2 min without playing any music. To avoid carryover effects, the music was played in the order calm, happy, sad and angry during EEG recordings. Each music passage was 45 s long, and to get same music number, we select 20 music passages in each music emotion group. To avoid volunteer fatigue, subjects rested for approximately 10–15 min between each block of 20 music passages in a given group. Every subject was asked to repeat the experiment 2 or 3 days later, yielding a total of 16 datasets. Due to failure of one trial, however, only 15 datasets were obtained.

**Figure 3:**
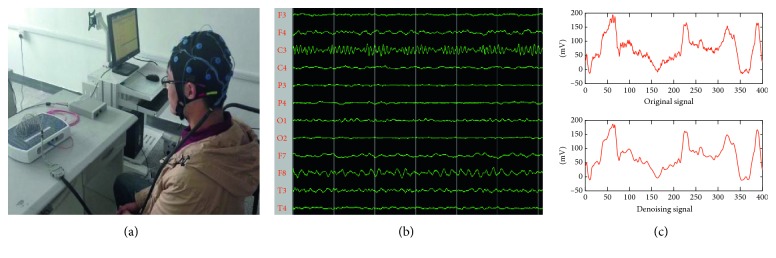
EEG acquisition equipment and filtering noise.

A NCC MEDICAL NCERP-P series EEG system and 24 electrodes EEG map was used for all recordings (Figure 3), and EEG electrodes were arranged according to the 10–20 international system. The average resistance of bilateral papillae reference and scalp recording electrodes was 5 k·Ohm, the sampling rate was 256 Hz, and the power frequency was 50 Hz. Before data processing, the eye power was filtered by ICA on EEG devices. The original signal is filtered through adaptive filtering to remove 50 Hz power frequency noise, and the final signal is obtained through wavelet filtering and preprocessing.

## 3. Data Analysis

### 3.1. Feature Extraction

**Figure 4:**
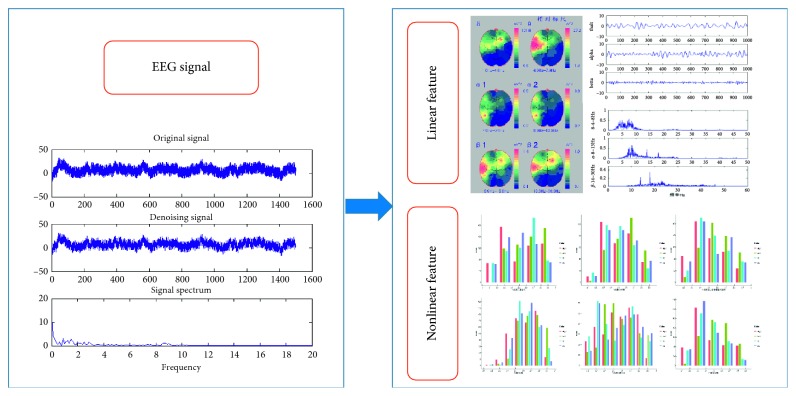
Features extracted from the EEG.

Many previous studies on EEG correlates of human emotion have examined only linear features [28–30]. However, developments in nonlinear dynamics have enhanced our understanding of the brain as a high-dimensional complex chaotic system, and nonlinear dynamic characteristics are now widely used in EEG research [31–33]. As shown in Figure 4, the EEG data acquired were first preprocessed and filtered for noise and the 50 Hz frequency signal filtered by adaptive filtering. The ICA signal was then filtered out to extract a 15 s EEG epoch for each music passage which is located in the middle of the 45 s music. This is because we want to avoid volunteers' mood swing at the beginning of the testing and fatigue at the end of the testing. So, the first and the last 15 s data were removed. We focused on electrodes FP1, FP2, F3, F4, F7, F8, Fz, C3, C4, T3, T4, and Pz as these positions are strongly related to emotion. The 18-dimensional linear features (peak, mean, variance, centre frequency, maximum power, and power sum of 3 frequency bands, theta, alpha, and beta) plus the 9-dimensional nonlinear dynamics features (singular spectral entropy, Lempel–Ziv complexity, spectral entropy, C0 complexity, maximum Lyapunov exponent, sample entropy, approximate entropy, K entropy, and correlation dimension) were extracted.

The linear features were expressed as following. Peak could be presented as

(1) $$\begin{array}{c} \text{Pe}=\max\left[x\left(n\right)\right]. \end{array}$$

where *x*(*n*) is the sampling EEG data.

Mean and variance are just the mean value and variance of *x*(*n*). Centre frequency could be expressed as

(2) $$\begin{array}{c} F_{a}=F_{\text{c}}\times \frac{f_{\text{s}}}{a}, \end{array}$$

where *a* is the scale value in wavelet transform and *f*_s_ is the sampling frequency; *F*_c_ is the wavelet centre frequency when the scale was 1; *F*_*a*_ was the centre frequency when the scale was a. If *r*(*k*) was the self-correlation function of EEG data *x*(*n*), maximum power could be defined as

(3) $$\begin{array}{c} \text{Pm}=\max\left[P\left(\omega \right)\right], \end{array}$$

in which,

(4) $$\begin{array}{c} P\left(\omega \right)=\overset{+\infty }{\underset{k=-\infty }{\sum }}r\left(k\right)e^{-j\omega k}. \end{array}$$

Power sum was the summation of *P*(*ω*).

The nonlinear features could be obtained according to the following references. Approximate entropy was presented by Pincus in 1983 [34]. It is a positive number and for EEG signals, the larger the value, the higher the complexity or the stronger the irregularity. Sample Entropy is an improvement of approximate entropy by Richman and Moorman [35], which can reduce the calculation error and improve the calculation accuracy. Correlation dimension is an important branch of fractal dimension and was proposed by Grassberger and Procaccia in 1983 [36]. K entropy was also called information dimension and was presented by Kolmogrov and was improved by Sinai. It can be expressed according to reference [37]. The higher the LZ complexity is, the more irregular the change of time series is. It indicates the rate at which a given time series increases with its length to cause a new pattern increasing. The new pattern shows a decreasing trend with the increase of time, which means that the change of original data series is slower. It can be expressed according to reference [38]. Whether the maximum Lyapunov exponent is greater than zero is the criterion to judge whether the system is chaotic or not, and it can be expressed according to reference [39]. The idea of the C0 complexity is to decompose the time series to be analyzed into random sequence and regular sequence. It can be expressed according to reference [40]. Singular spectrum analysis is to reconstruct the delay of one-dimensional EEG time series into multidimensional phase space. After singular value decomposition, the importance of decomposed quantities is determined according to the order of energy [41]. The spectral entropy can be expressed according to [42]. In the procedure, firstly, the signal is transformed by Fourier transform, then the power distribution of the signal is calculated and the unit power is normalized.

### 3.2. Feature Selection

The CFS method, which is widely used for feature selection and data cleaning, comprehensively evaluates the correlations between features and classifications as well as the redundancy between features [43, 44]. The core idea of this method is to remove redundant features and select unique class-related features from the original feature set using correlation analysis [45]. Briefly, CFS calculates the correlations between features as well as between features and categories of feature concentration. The calculation formula is shown below:

(5) $$\begin{array}{c} \text{Merit}=\frac{k\bar{r_{\text{cf}}}}{\sqrt{k+k\left(k-1\right)\bar{r_{\text{ff}}}}}. \end{array}$$

Merit is an evaluation of the characteristic subset *s*, where *s* contains *k* characteristics, $\bar{r_{\text{cf}}}$ represents the average correlation between feature *f*(*f* ∈ *S*) and category *c*, and $\bar{r_{\text{ff}}}$ represents the average relativity value between features. Formula (5) shows that for the feature subset *s*, when the correlations between each feature and class label were greater, and the correlations between each two features were smaller, the value of merit would be much greater, and it means that the feature set *s* was the better one.

The correlation between the features can be calculated using the information gain method. Assuming that *y* is a possible value of attribute *Y*, then the entropy of *Y* is calculated as

(6) $$\begin{array}{c} H\left(Y\right)=-\underset{y\in Y}{\sum }p\left(y\right)\;\log_{2}\left(p\left(y\right)\right). \end{array}$$

If a certain property *X* is known, the method to calculate the entropy of *Y* under *X* conditions is

(7) $$\begin{array}{c} H\left(Y\;|\;X\right)=-\underset{x\in X}{\sum }p\left(x\right)\underset{y\in Y}{\sum }p\left(y\;|\;x\right)\;\log_{2}\left(p\left(y\;|\;x\right)\right). \end{array}$$

The additional information that feature *X* contributes to *Y* is called information gain. The correlation between information gain and two features is positive. Information gain is defined as

(8) $$\begin{array}{c} H\left(Y\right)-H\left(Y\;|\;X\right). \end{array}$$

Since information gain is a measurement method of symmetry, it must be normalized. To this end, we used the following formula:

(9) $$\begin{array}{c} U_{XY}=2.0\times \frac{H\left(Y\right)-H\left(Y\;|\;X\right)}{H\left(Y\right)+H\left(X\right)}. \end{array}$$

The correlation coefficient describes the strength of correlation between two variables, with values closer to 1 indicating a stronger correlation.

A greedy progressive search algorithm was used to generate candidate feature subsets from feature sets. Using this algorithm, feature selection produces a feature sequence that is sorted by the degree of correlation *f*_1_, *f*_2_, *f*_3_,…, *f*_27_, then uses the classifier to identify the feature subset (*f*_1_), (*f*_1_, *f*_2_), (*f*_1_, *f*_2_, *f*_3_), (*f*_1_, *f*_2_, *f*_3_, *f*_4_), (*f*_1_, *f*_2_, *f*_3_, *f*_4_,…*f*_25_, *f*_26_).

As shown in Table 3, for every feature, we calculated the average Merit value of every volunteer. From the result, if we set the threshold as 0.1, the average Merit value of feature 4, 8, 10, 14, 16, 17, 20, 21, 22, 23, 25, 26 are higher than the threshold while other features were much lower. So, they were selected as the influential features which were agreed with the data in Table 4.

### 3.3. Classifier Verification

**Figure 5:**
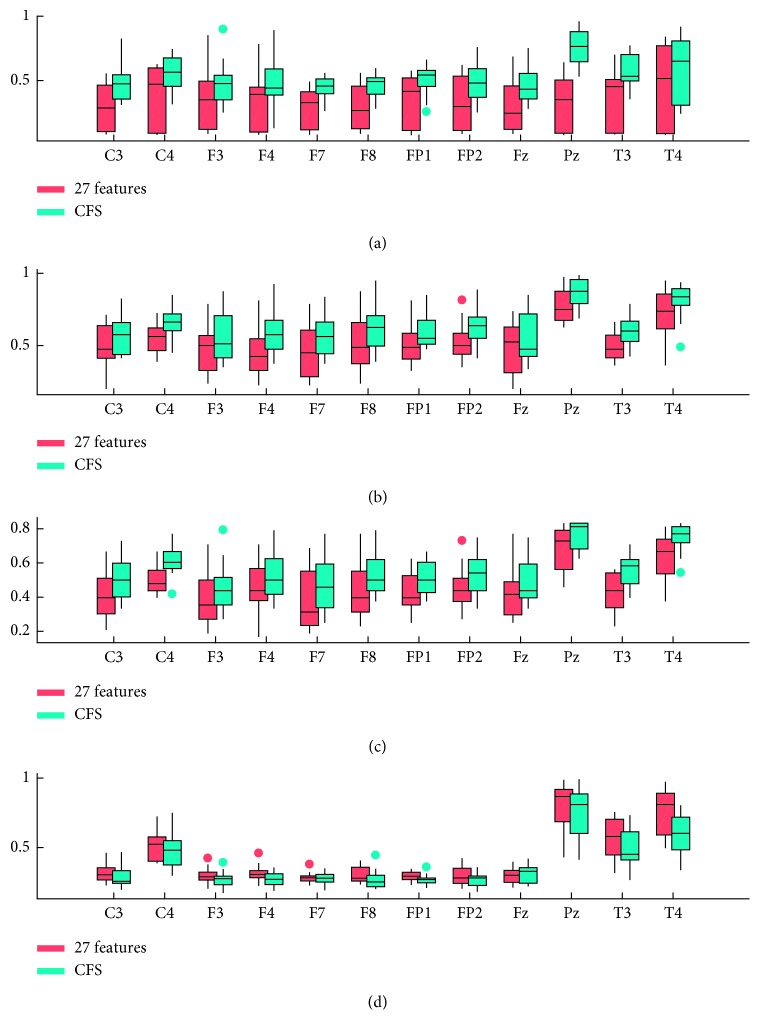
The recognition rates of SVM, C4.5, BP, and LDA classifiers before and after dimension reduction. (a) Feature reduction recognition rate verified by SVM. (b) Feature reduction recognition rate verified by C4.5. (c) Feature reduction recognition rate verified by BP. (d) Feature reduction recognition rate verified by LDA.

SVM, decision tree, and neural network are the most common classification methods used in machine learning. In this study, SVM, C4.5, BP neural network, and LDA were used to verify the recognition rate (accuracy) after feature selection. We used the method of 10% cross validation, 10% of data is the training data, then took the mean value of 100 times repetitions to identify the correct rate as the recognition rate. Finally, as shown in Figure 5, for every subfigure, the horizontal axis is the electrodes and the vertical axis is the recognition rate. For every classification method, using Pz, T3, and T4 electrodes' data, we can get much higher recognition rates than using other electrodes. So, these 3 electrodes were chosen in the classification procedure. Moreover, to compare the results in details, the recognition accuracy values using the 3 electrodes' data through the four classification methods are shown in Table 5.

According to classification results, the recognition rates of the features selected by CFS (feature subset) were greater than the recognition rates before dimension reduction using SVM, C4.5, and BP. But for LDA method, the situation was opposite. According to previous validation studies, LDA is a robust emotion classifier [46]. But in the current study, BP, SVM, and C4.5 achieved good classification accuracy.

**Figure 6:**
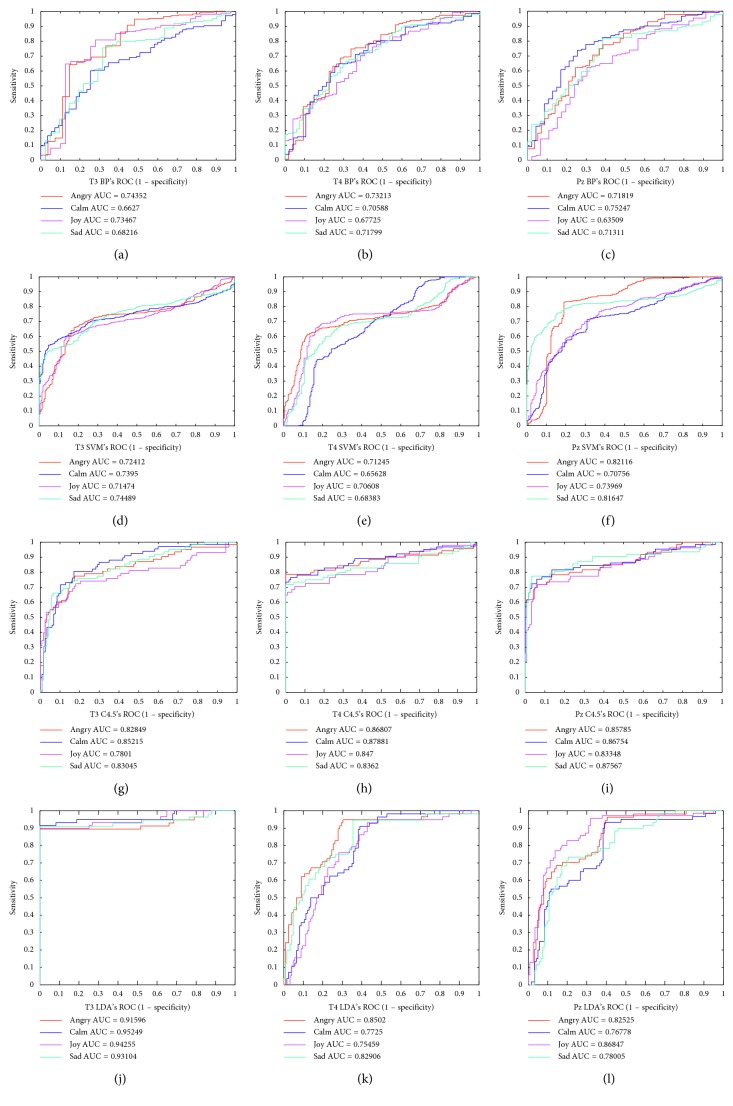
The ROC curves of SVM, C4.5, BP, and LDA classifiers.

In order to evaluate the classifier performance, the ROC curves of four different classifiers are shown in Figure 6. The ROC curve of C4.5 and LDA classifier is good, and the AUC average value is larger, so the performance of LDA and C4.5 classifier is better than that of BP and SVM.

For statistical feature analysis, two samples of *t*-tests were performed on the different emotional features and different electrodes (at significance level *p*=0.05). Then, each feature of each electrode was tested by paired *t*-test, and the number of features rejected according to the null hypothesis was recorded.  Table 6 examines whether the features of the same electrode are irrelevant when different emotions are stimulated, and the values in the table indicate the number of *P* values bigger than 0.05. From the *t*-test results in Table 6, the number of unrelated features was greater for electrodes T4 and Pz than for any other electrode pair.

### 3.4. Feature Test

**Figure 7:**
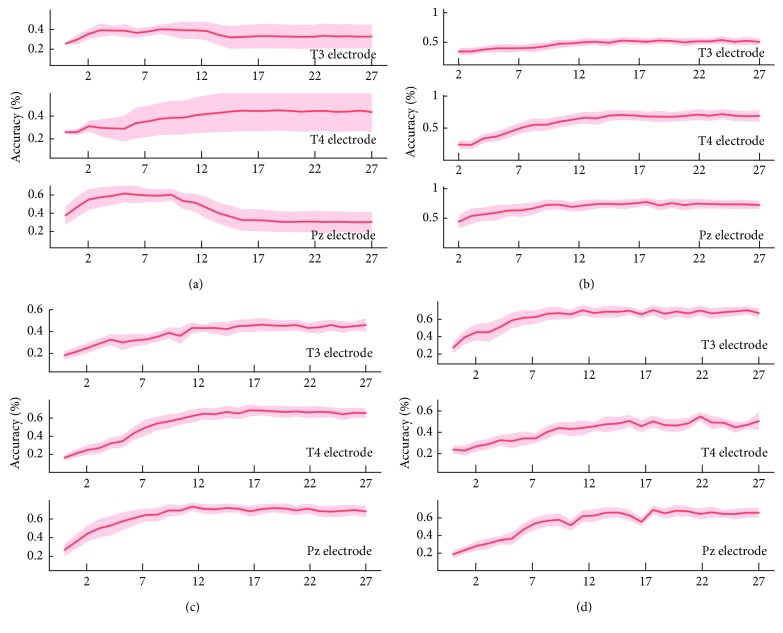
Recognition accuracies of different feature subset from electrodes T3, T4, and Pz verified by different classifiers. (a) Recognition accuracy of different feature subsets of T3, T4, and Pz verified by SVM. (b) Recognition accuracy of different feature subset of T3, T4, and Pz verified by C4.5. (c) Recognition accuracy of different feature subset of T3, T4, and Pz verified by BP. (d) Recognition accuracy of different feature subset of T3, T4, and Pz verified by LDA.

The analysis above indicates that the features selected by CFS are more effective for discrimination than the original features. To identify the most effective features in the original feature set, the features in the optimal feature subset, which means the feature set contains *n* features selected by CFS for one volunteer, were analyzed. Since the optimal feature subset selected by CFS a little differs by volunteer, it is necessary to consider the optimal recognition rate which is get using optimal feature subset, for all volunteers. Therefore, we extracted the recognition rate of all feature subsets for all subjects and verified the best feature subset for most subjects. From the analysis shown in Figure 7, when the feature number *n* changes from 2 to 27, electrodes T4 and Pz were the most effective for recognition of music-evoked emotions. Moreover, recognition accuracy was better of C4.5 than other classification methods, and the accuracies of T4 and Pz electrodes are much better than that of P3. The data corresponding to Figure 7 are shown in Table 7.

**Figure 8:**
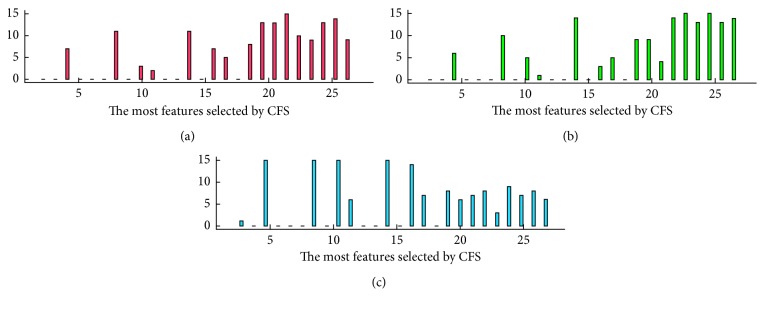
Frequency histograms of features from electrodes T3, T4, and Pz.

This recognition analysis using signals from different electrodes and different classifiers indicated that recognition accuracy was not improved so much or was even reduced when the selected feature subset had more than 10 dimensions, regardless of the recognition algorithm or EEG channel used. The top 10-dimensional feature subsets of each music group in the 15 datasets were selected to construct a frequency distribution histogram. In the histogram, a more effective feature has higher frequency to be selected by CFS. As shown in Figure 8, for three electrodes, there are 15 volunteers' experiment data of different features in frequency domain. The total chosen times of every feature by CFS for every volunteer are shown in Table 4. We chose the features whose chosen time was higher than 20 as the features correlated with the class labels closely, such as feature 4 (Alpha centre frequency), 8 (Theta average value), 10 (Theta centre frequency), 14 (Beta average value), 16 (Beta centre frequency), 17 (Beta maximum power), 20 (entropy of K), 21 (approximate entropy), 22 (the maximum Lyapunov exponent), 23 (the complexity of C0), 25 (the spectral entropy), and 26 (Lempel–Ziv complexity). The above features included 6-dimensional linear features, and 6-dimensional nonlinear features were the final selected optimal feature subset. This represents the characteristic feature combination most effective for most subjects.

## 4. Experiments and Analysis

### 4.1. Linear Features

We found that the beta wave accounted for the largest proportion of linear features selected by CFS, consistent with previous research on emotion recognition [47]. Of the linear features, the band centre frequency was also of obvious importance, possibly because the centre frequency best represents and distinguishes the band.

In this study, EEGLAB was used to analyse the linear features. The steps are as follows. First, the wavelet was used to process the frequency-band divisions (theta, alpha, and beta) of the 15 datasets. Then, the EEG data acquired during music evoking the four emotions, and the associated frequency bands were superimposed on the average. Finally, EEGLAB was imported and brain topographic maps constructed for each emotion using the 12 emotion-related electrodes.

**Figure 9:**
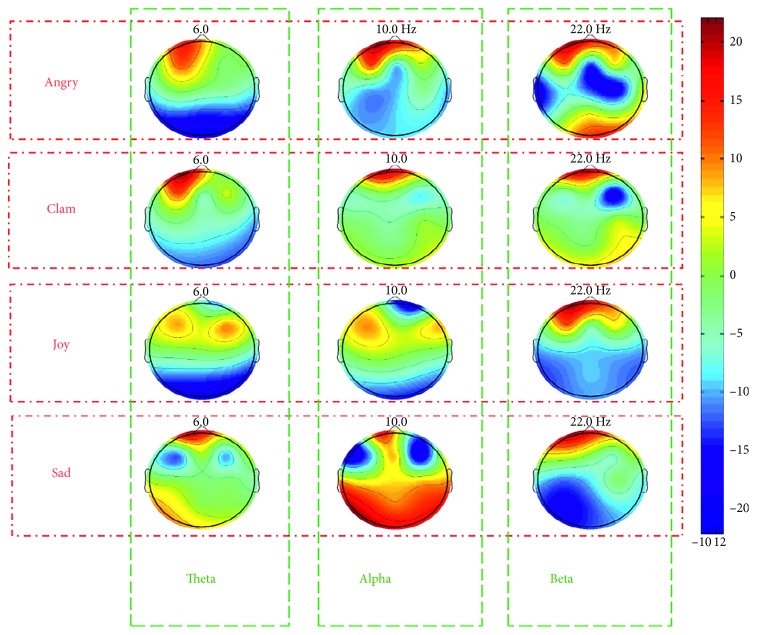
Three distinct frequency-band topographic maps distinguishing the emotions calmness, happiness, sadness, and anger evoked by music.

As shown in Figure 9, when the subject was listening to angry or quiet music, band energy was higher in the frontal area. When listening to joyful music, theta and alpha band energy was higher in the occipital cortex region, while beta band energy was higher at the forehead. When listening to sad music, the alpha wave exhibited a wider activity range. Comparing these activity patterns, the alpha band appears to change mostly with emotion evoked by music, suggesting that the alpha band is more active when listening to emotionally evocative music.

### 4.2. Nonlinear Features

**Figure 10:**
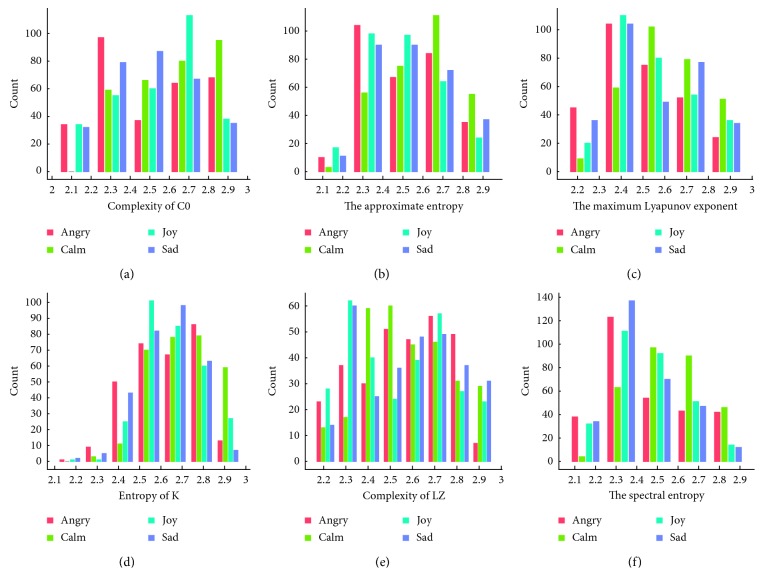
Nonlinear characteristic frequency distribution of the Pz electrode.

To analyse the nonlinear characteristics of the selected feature set, as shown in Figure 10, we constructed a frequency histogram of the selected features and studied the relationships between different emotions and nonlinear characteristics.

For the Pz electrode, from the distribution histogram of the 6 features in Figure 10, we can find some relationships between it and the emotion classifications. The value of “angry” was mostly distributed in the lower numerical segment in the histogram of maximum Lyapunov exponent. It is the same as the histogram of spectral entropy. But for “calm”, numerical distribution differences were not so obvious except in complexity of C0 histogram. The value of “joy” was mostly distributed in the higher numerical segment in the histogram of complexity of C0, entropy of K, and spectral entropy compared with “angry”. The value of “sad” was mostly distributed in the higher numerical segment in the histogram of approximate entropy and spectral entropy.

**Figure 11:**
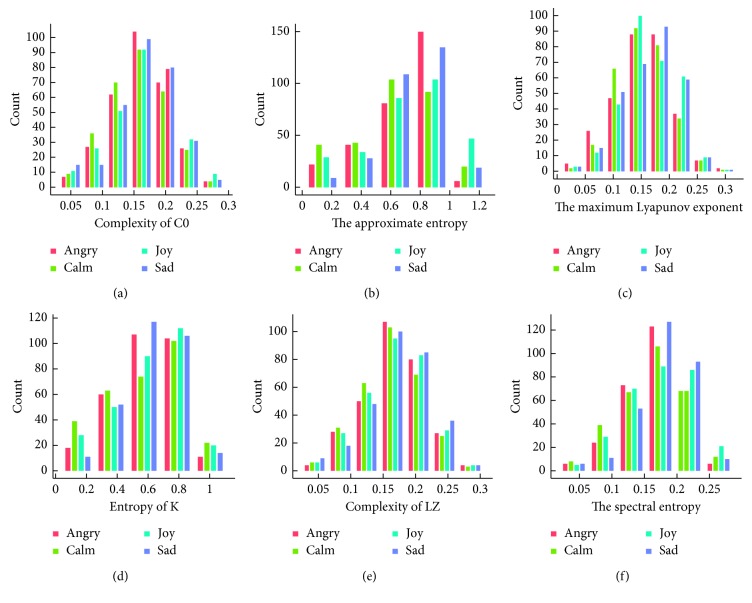
Nonlinear characteristic frequency distribution of the T3 electrode.

For the T3 electrode, as shown in Figure 11, we can find some differences between the histograms of “angry” and “sad” according to approximate entropy, entropy of K, and spectral entropy. But, the relationships between features and emotions were less obvious than for electrodes Pz and T4.

**Figure 12:**
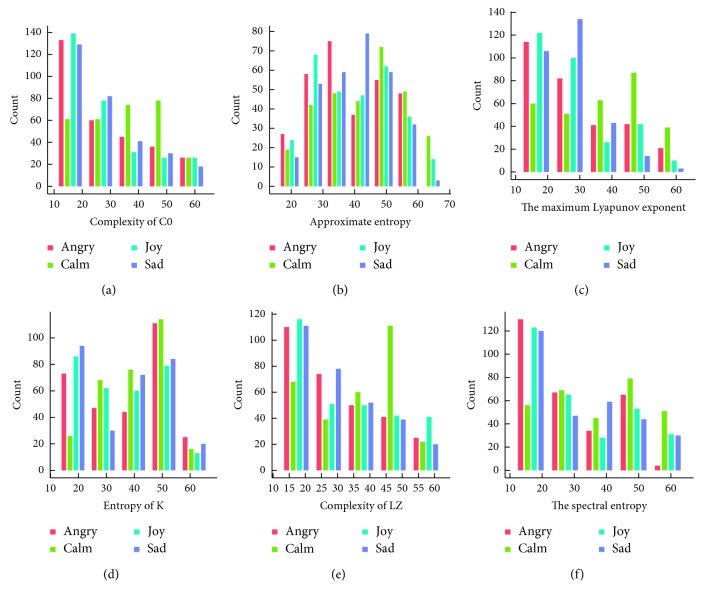
Nonlinear characteristic frequency distribution of the T4 electrode.

For the T4 electrode, from the distribution histogram of the 6 features in Figure 12, we can also find some relationships between it and the emotion classifications. The value of “angry” was mostly distributed in the lower numerical segment in the histogram of complexity of C0, maximum Lyapunov exponent, complexity of LZ, and spectral entropy. But for “calm”, numerical distribution differences were also not so obvious except in complexity of LZ histogram. The value of “joy” was mostly distributed in the higher numerical segment in the histogram of complexity of C0, maximum Lyapunov exponent, complexity of LZ, and spectral entropy. The value distribution of “sad” was very similar to the distribution of “joy”, except some *s* small differences in approximate entropy and entropy of K.

By the comparison of nonlinear features' value distribution histogram, some differences could be found between the four emotion states. Some differences were obvious such as “angry,” “joy,” and “sad”. But for “calm,” the differences were not so obvious.

### 4.3. Examination and Repeatability

**Figure 13:**
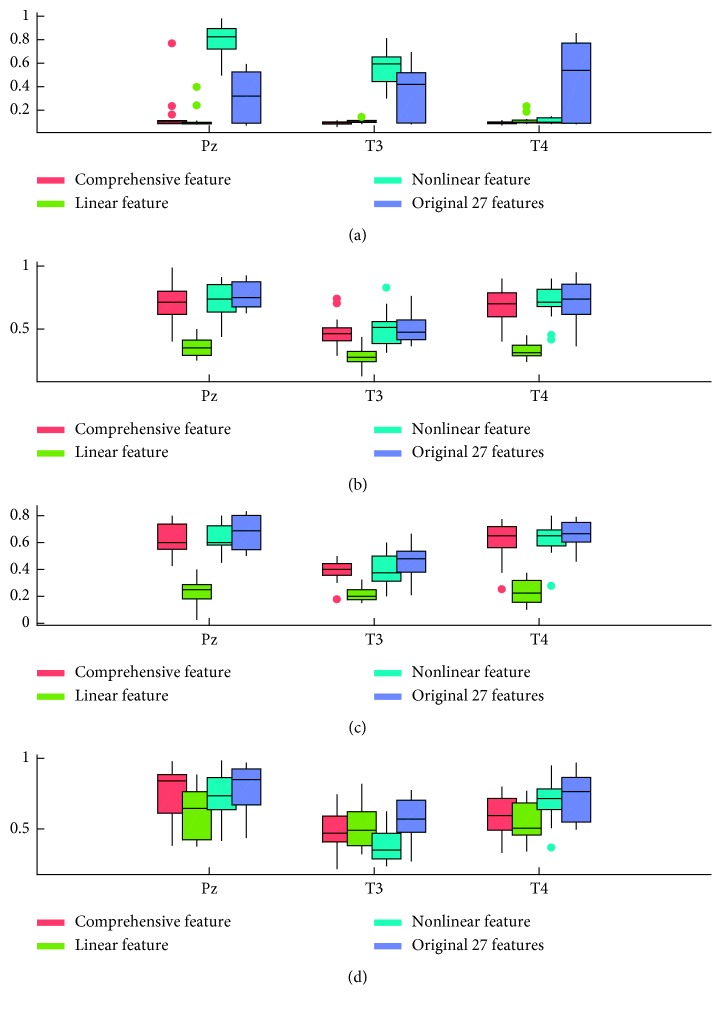
Recognition accuracy comparison among feature subsets. (a) Feature reduction recognition rate verified by SVM. (b) Feature reduction recognition rate verified by C4.5. (c) Feature reduction recognition rate verified by BP. (d) Feature reduction recognition rate verified by LDA.

We then compared emotion recognition accuracy among the 6-dimensional linear features, 6-dimensional nonlinear features, the selected 12-dimensional features, and the 27-dimensional features using different algorithms. The recognition rate was higher for nonlinear features than linear features for all algorithms, possibly because differences in nonlinear EEG features are larger than the deviations of frequency features within the same frequency band, such as the mean values of centre frequency and maximum frequency. Therefore, nonlinear features may be more suitable for classification of mood evoked by music. The selected features were then compared with the original 27-dimensional features. Accuracy was not significantly higher than that with the original set, possibly because these features were selected based on a comparison of all datasets across subjects and were not the best set for any individual. However, the smaller number of dimensions with equivalent accuracy indicates that redundant features were removed. Therefore, the 12-dimensional features selected can be regarded as the main EEG features distinguishing the emotions calm, anger, joy, and sadness evoked by music. So, we selected the nonlinear features of Pz electrodes as the best feature set for emotion recognition. The results are shown in Figure 13.

To test the repeatability of the classification effectiveness of the selected feature set, we used the EEG signal of the 15 volunteers as the repeatability examination data source. The classification procedures were repeated for 10 times for each volunteer. For every one, the EEG data was selected in four emotion statures including joy, sad, calm, and angry. That was, for every emotion of every volunteer, 8 randomly selected EEG data were employed as testing data, and the other 72 EEG data were used as the training data. The classification results are listed in Table 8. It showed that the average recognition rates were all higher than 85% and the nonlinear features of Pz electrode were effective for emotion recognition.

The DEAP database has also been tested in this paper. We used the same data label as the article [1]. The score of valence and arousal dimension 1–3 is regarded as low group and 7–9 is high group, and finally, more than 20 EEG data evoked by videos were selected for every volunteer. The 12-dimension features mentioned in this paper were employed as the feature set. The result is that classification accuracy is higher than the statistical characteristics mentioned in the article. And as shown in Table 9, for C4.5 classifier, the correct classification rate is 84.91% and 89.65% for valence and arousal.

## 5. Conclusions

This study analyzed the linear and nonlinear characteristics of EEG signals recorded during music evoking distinct emotional responses (calm, joy, sadness, and anger) and identified those features most effective for accurate EEG-based recognition of emotion. The EEG characteristics of 12 EEG electrodes yielded 27 dimensions each. Statistical analysis revealed that electrodes T3, T4, and Pz were most likely to be associated with the music stimulus. The CFS method was used to identify those features most effective for EEG-based emotion recognition without redundancy. We then used different classifiers to test whether the selected feature set was more accurate than the original feature data. The results can be summarised as follows:

1. The algorithms C4.5 are more effective for emotion classification of EEG signals than LDA, BP neural network, and SVM.
2. We used brain topographic maps and frequency distribution histograms to identify the optimal subset of linear and nonlinear features of the three electrodes and identified six-dimensional linear features (centre frequency of the alpha band, mean theta band, centre frequency of the theta wave, mean delta band, centre frequency of the delta band, and maximum power of the delta wave) plus six-dimensional nonlinear features (entropy of K, approximate entropy, maximum Lyapunov exponent, C0 complexity, spectral entropy, and LZ complexity). These dimensions may be the most representative features for the classification of music-evoked emotions.
3. We compared discrimination accuracy among the different combinations of linear and nonlinear features and found that the nonlinear features were more effective. Finally, the 12 selected dimensional features were as accurate as the original 27 dimensions, indicating that redundant features were eliminated. Then the classification results of 10 times randomly examinations, it could be concluded that the selected 6 nonlinear features of Pz are more effective than other features when used as the key feature set to classify human emotion statues.

## Figures and Tables

**Table 1:** Homogeneity test of variance.

Pairs	Levene statistic	df1	df2	Saliency
sad_arousal-clam_arousal	2.445^a^	7	12	0.083
sad_arousal-joy_arousal	2.905^b^	7	12	0.050
sad_arousal-angry_arousal	2.749^c^	7	12	0.054
clam_arousal-joy_arousal	1.036^a^	4	14	0.423
clam_arousal-angry_arousal	1.392^b^	4	14	0.287
joy_arousal-angry_arousal	0.473^a^	4	14	0.755
sad_valence-clam_valence	0.398^a^	5	13	0.842
sad_valence-joy_valence	2.512^b^	5	13	0.084
sad_valence-angry_valence	2.307^c^	5	13	0.104
clam_valence-joy_valence	1.219^a^	6	13	0.357
clam_valence-angry_valence	0.969^b^	6	13	0.483
joy_valence-angry_valence	2.516^a^	3	11	0.058

a, b, and c are the ignored abnormal values

**Table 2:** Variance analysis.

Pairs	Sum of squares	df	Mean of square	*F*	Saliency
sad_arousal ∗ clam_arousal	1.195	7	0.171	5.898	0.033
sad_arousal ∗ joy_arousal	1.377	11	0.125	7.597	0.026
sad_arousal ∗ angry_arousal	2.326	14	0.166	10.661	0.015
clam_arousal ∗ joy_arousal	0.354	11	0.032	521.398	0.001
clam_arousal ∗ angry_arousal	0.654	14	0.047	23.641	0.001
joy_arousal ∗ angry_arousal	2.46	14	0.176	10.649	0.018
sad_valence ∗ clam_valence	0.593	8	0.074	231.87	0.001
sad_valence ∗ joy_valence	1.037	8	0.13	12.58	0. 015
sad_valence ∗ angry_valence	0.539	10	0.054	11.042	0.018
clam_valence ∗ joy_valence	1.56	10	0.156	22.03	0.001
clam_valence ∗ angry_valence	0.957	10	0.096	22.464	0.001
joy_valence ∗ angry_valence	2.025	10	0.203	1.16	0.404

**Table 3:** The differences of average value *U*_*xy*_ for every feature.

No.	Feature	*U*_*xy*_
1	Alpha peak	0.042
2	Alpha average value	0.061
3	Alpha variance	0.035
4	Alpha centre frequency	**0.146**
5	Alpha maximum power	0.024
6	Alpha power sum	0.021
7	Theta peak	0.019
8	Theta average value	**0.127**
9	Theta variance	0.031
10	Theta centre frequency	**0.189**
11	Theta maximum power	0.072
12	Theta power sum	0.012
13	Beta peak	0.031
14	Beta average value	**0.194**
15	Beta variance	0.014
16	Beta centre frequency	**0.124**
17	Beta maximum power	**0.101**
18	Beta power sum	0.023
19	Singular spectral entropy	0.055
20	Entropy of K	**0.202**
21	Approximate entropy	**0.138**
22	Maximum Lyapunov exponent	**0.129**
23	Complexity of C0	**0.121**
24	Sample entropy	0.061
25	Spectral entropy	**0.183**
26	Lempel–Ziv complexity	**0.211**
27	Correlation dimension	0.074

**Table 4:** Accuracies for different dimension features subset across different electrode.

No.	Feature	Selected times	Correlation degree
1	Alpha peak	0	Low
2	Alpha average value	2	Low
3	Alpha variance	0	Low
4	Alpha centre frequency	28	High
5	Alpha maximum power	0	Low
6	Alpha power sum	0	Low
7	Theta peak	0	Low
8	Theta average value	41	High
9	Theta variance	0	Low
10	Theta centre frequency	26	High
11	Theta maximum power	7	Low
12	Theta power sum	0	Low
13	Beta peak	0	Low
14	Beta average value	40	High
15	Beta variance	0	Low
16	Beta centre frequency	27	High
17	Beta maximum power	21	High
18	Beta power sum	0	Low
19	Singular spectral entropy	19	Low
20	Entropy of K	30	High
21	Approximate entropy	27	High
22	Maximum Lyapunov exponent	35	High
23	Complexity of C0	31	High
24	Sample entropy	18	Low
25	Spectral entropy	39	High
26	Lempel–Ziv complexity	40	High
27	Correlation dimension	19	Low

**Table 5:** The classification accuracy through the 4 methods using 27 features and the CFS feature set.

Methods	Feature set	Electrode	Recognition rate (%)
SVM	27 original features	Pz	47.68
		T3	51.22
		T4	57.49
	Feature set selected by CFS	Pz	75.42
		T3	55.83
		T4	63.74

C4.5	27 original features	Pz	72.24
		T3	52.56
		T4	73.32
	Feature set selected by CFS	Pz	85.46
		T3	63.78
		T4	80.95

BP	27 original features	Pz	79.63
		T3	54.64
		T4	72.27
	Feature set selected by CFS	Pz	82.55
		T3	62.96
		T4	78.71

LDA	27 original features	Pz	92.23
		T3	63.54
		T4	88.65
	Feature set selected by CFS	Pz	89.78
		T3	56.89
		T4	70.43

**Table 6:** The unrelated numbers of 12 electrodes of different emotions using paired *t*-test (*p* > 0.05).

Electrode	FP1	FP2	F3	F4	F7	F8	Fz	C3	C4	T3	T4	Pz
Anger-calm	146	135	139	144	136	112	138	130	125	94	168	152
Anger-joy	132	116	107	130	109	105	115	126	76	92	157	142
Anger-sad	99	126	83	99	100	97	100	117	66	67	120	127
Calm-joy	126	124	101	116	97	119	121	109	99	80	124	129
Calm-sad	161	151	127	138	151	133	137	149	126	107	179	164
Joy-sad	112	127	94	111	116	110	103	127	89	103	162	145
Total	776	779	651	738	709	676	714	758	581	543	910	859

**Table 7:** Accuracies for different dimension features subset across different electrode.

Feature number	2	5	10	15	20	25
SVM (%)						
T3	25.35	40.96	42.95	37.63	38.64	38.99
T4	24.75	30.67	41.66	42.86	43.52	46.23
Pz	40.67	61.53	60.97	41.63	39.94	38.01

C4.5 (%)						
T3	37.56	40.82	50.67	56.32	55.77	56.18
T4	36.64	57.63	73.05	74.57	74.69	76.77
Pz	57.81	67.74	75.19	78.63	77.88	78.90

BP (%)						
T3	20.23	32.74	40.12	45.64	47.57	48.92
T4	19.27	38.69	62.45	63.74	62.29	64.67
Pz	27.54	58.78	73.13	71.62	70.87	71.33

LDA (%)						
T3	25.63	59.67	64.78	65.14	64.97	65.34
T4	21.78	32.71	45.91	50.83	47.63	46.92
Pz	20.79	38.98	58.67	62.38	63.00	62.47

**Table 8:** Repeatability of the classification effectiveness.

1	2	3	4	5	6	7	8	9	10	Ave
100	87.5	87.5	87.5	87.5	87.5	100	87.5	100	75	90
100	100	100	100	87.5	100	87.5	87.5	87.5	75	92.5
100	87.5	87.5	100	87.5	100	100	100	100	87.5	95
87.5	87.5	75	87.5	87.5	87.5	87.5	87.5	87.5	100	87.5
87.5	87.5	87.5	100	100	100	100	87.5	87.5	100	93.75
100	87.5	87.5	100	87.5	75	87.5	100	87.5	100	91.25
100	100	100	87.5	100	100	100	100	87.5	100	97.5
87.5	87.5	75	87.5	87.5	87.5	87.5	87.5	87.5	87.5	86.25
87.5	100	75	87.5	87.5	100	87.5	87.5	75	87.5	87.5
100	100	100	100	87.5	100	100	100	100	100	98.75
87.5	100	100	87.5	100	87.5	100	87.5	100	87.5	93.75
87.5	87.5	75	87.5	87.5	87.5	87.5	87.5	75	87.5	85
87.5	87.5	87.5	75	87.5	87.5	75	87.5	87.5	87.5	85
100	87.5	100	87.5	87.5	87.5	100	87.5	87.5	87.5	91.25
87.5	87.5	87.5	100	87.5	100	87.5	87.5	87.5	100	91.25

**Table 9:** Recognition accuracy rate of the DEAP database.

Method	Valence (%)	Arousal (%)
SVM	73.45	80.20
BP	74.67	77.92
C4.5	84.91	89.65
LDA	72.33	76.68

## Data Availability

The music stimuli used in this study are from a database of 1000 songs, which was selected by Mohammad Soleymani and colleagues of the University of Geneva for emotion analysis from the Free Music Archive (http://freemusicarchive.org/).
